# Evaluation of Protective Immune Responses Induced by Recombinant TrxLp and ENO2 Proteins against* Toxoplasma gondii* Infection in BALB/c Mice

**DOI:** 10.1155/2016/3571962

**Published:** 2016-10-10

**Authors:** Meng Wang, Xiao-Yu Yang, Nian-Zhang Zhang, De-Lin Zhang, Xing-Quan Zhu

**Affiliations:** State Key Laboratory of Veterinary Etiological Biology, Key Laboratory of Veterinary Parasitology of Gansu Province, Lanzhou Veterinary Research Institute, Chinese Academy of Agricultural Sciences, Lanzhou, Gansu 730046, China

## Abstract

*Toxoplasma gondii* is an obligate intracellular parasitic protozoan that can infect almost all species of warm-blooded animals. As any chemical-based drugs could not act against the tissue cyst stage of* T. gondii*, vaccination may be one of the ideal control strategies. In the present study, two new vaccine candidates, named TgENO2 and TgTrxLp, were purified from* Escherichia coli* with pET-30a(+) expression system and then were injected into BALB/c mice to evaluate the protective efficacy against acute and chronic toxoplasmosis. The results showed that both the recombinant proteins, either alone or in combination, could elicit strong humoral and cellular immune responses with a higher level of IgG antibodies, IFN-*γ*, IL-2, CD4^+^, and CD8^+^ T cells as compared to those in mice from control groups. After acute challenge with tachyzoites of the GJS strain, mice immunized with rTgTrxLp (8 ± 2.77 d), rTgENO2 (7.4 ± 1.81 d), and rTgTrxLp + rTgENO2 (8.38 ± 4.57 d) proteins showed significantly longer survival time than those that received Freund's adjuvant (6.78 ± 2.08 d) and PBS (6.38 ± 4.65 d) (*χ* ^2^ = 9.687, df = 4, *P* = 0.046). The protective immunity of rTgTrxLp, rTgENO2, and rTgTrxLp + rTgENO2 proteins against chronic* T. gondii* infection showed 69.77%, 58.14%, and 20.93% brain cyst reduction as compared to mice that received PBS. The present study suggested that both TgENO2 and TgTrxLp were potential candidates for the development of multicomponent vaccines against toxoplasmosis.

## 1. Introduction

*Toxoplasma gondii* is a worldwide prevalent pathogen in all the warm-blooded animals including humans [1, 2].* T. gondii* infection in immune-competent individuals is rarely symptomatic. However, the infection occurring in the fetus or immunocompromised patients (HIV patients) could result in severe diseases or even death [3–5]. Infection of domestic animals with the parasite can cause substantial economic losses and also pose a considerable threat to public health [6–10].

The infection begins from ingestion of oocysts or cysts of* T. gondii*. Once the tachyzoites invade into the intestinal epithelial cells, the parasites rapidly proliferate by intracellular endodyogeny [1]. During the division of tachyzoites,* T. gondii* enolase (TgENO2) exhibits robust nuclear labeling with the ability to bind promoters and to regulate the gene expression [11–13].

Subsequent to the infection of the intestinal epithelial cells,* T. gondii* disseminate throughout the organism. During the migration, the protozoan is exposed to reactive oxygen species (ROS) generated by inflammatory cells.* T. gondii* evolved a series of antioxidant proteins to relieve the oxidative stress produced by the host immune system. Thioredoxin (Trx) is one of the important antioxidants against the ROS destruction that can convert hydrogen peroxide (H_2_O_2_) to water [14–16]. The thioredoxin-like protein (TrxL) in* T. gondii* was discovered as a component of a microtubule-associated complex, but not directly interacting with the microtubules which would support numerous vital cellular functions in eukaryotes [17].

In our previous study, both of the enolase and Trx proteins were identified in* T. gondii* excreted/secreted antigens (TgESA) from mice enterocelia after being infected by the parasite (unpublished data). The crude ESAs of* T. gondii* are considered attractive vaccine candidates that have been widely studied in animal models [18–20]. However, it is yet to be clarified whether TgENO2 and TgTrxLp, as the constituents of TgESAs, could induce protective immune responses against* T. gondii* infection in the mouse model. In the present study, the immunogenicity of TgENO2 and TgTrxLp recombinant proteins was examined in mice. Furthermore, the immunoprophylaxis efficacy against acute and chronic toxoplasmosis was also estimated.

## 2. Materials and Methods

### 2.1. Animals

The specific-pathogen-free (SPF) grade BALB/c and Kunming mice (6–8 weeks old) were purchased from Lanzhou Veterinary Research Institute Laboratory Animal Center (Lanzhou, China). All animals were strictly handled according to the Good Animal Practice Requirements of the Animal Ethics Procedures and Guidelines of the People's Republic of China. The present study was approved by the Animal Ethics Committee of Lanzhou Veterinary Research Institute, Chinese Academy of Agricultural Sciences (Approval no. LVRIAEC2012-011).

### 2.2. Parasites

Tachyzoites of* T. gondii* GJS (Genotype DB9#) strain were maintained in Kunming mice by a series of intraperitoneal infections and obtained from the peritoneal exudates, followed by purification by centrifugation, as described by Qu et al. [21, 22]. Kunming mice infected with the low virulent PRU strain (Genotype II) were used to collect the toxoplasma cysts that were orally passaged by infection of the brain homogenate.

### 2.3. Prokaryotic Expression of TgTrxLp and TgENO2 Proteins and Purification

The full-length coding sequences of the* TgTrxLp* (GenBank access number XM_002369703.1) and* TgENO2* (GenBank accession number AF123457.1) genes were amplified using one-step reverse transcription-PCR (RT-PCR), following the manufacturer's instructions (Takara, China). The primers for the amplification of* TgTrxLp* fragment were 5′-GG*GGTACC*ATGGCGCCTCTGCGTGTGTGCGCGTTC-3′ (forward) and 5′-CC*AAGCTT*TTACAGTTCGTCCTTCTTGTCTGCCTT-3′ (reverse) and for* TgENO2* fragment were 5′-CG*GAATTC*ATGGTGGCCATCAAGGACATCACTGCT-3′ (forward) and 5′-CC*AAGCTT*TTAGTTGGGATGGCGGAAGCCAGCGCC-3′ (reverse). The restriction sites induced in the two pairs of primers were italicized.

Each RT-PCR product was purified (Tiangen, China) and ligated into the prokaryotic expression vector pET-30a(+)* via* the respective restriction sites, forming the recombinant plasmids, pET-TrxLp and pET-ENO2, respectively. Subsequently, the two recombinant plasmids were transformed into* Escherichia coli* strain BL21(DE3) and induced with 1.0 mmol/L isopropyl *β*-d-1-thiogalactopyranoside (Sangon, China), shaking for 6 h at 30°C. The rTgTrxLp and rTgENO2 proteins were purified on a Ni^2+^ column (Novagen, USA) following ultrasonic bacterial lysis on the ice. The purified protein samples were analyzed with sodium dodecyl sulfate-polyacrylamide gel electrophoresis (SDS-PAGE).

### 2.4. Western Blot Analysis

The proteins were resolved on SDS-PAGE, and the purified proteins were transferred to nitrocellulose (NC) membranes (Pall, USA). Then, the membranes were blocked with 5% bovine serum albumin (BSA) in PBST (0.05% Tween-20 in PBS) at room temperature (RT). After 1 h, the membrane was washed 4 times with PBST. The swine sera against* T. gondii* that were collected at 60 days after the onset of symptoms, as described previously [23], were diluted 1 : 1000 as the first antibody probed on the membranes for 1 h at RT. Then, the membranes were washed 4 times with PBST and incubated with horseradish peroxidase- (HRP-) conjugated goat anti-pig IgG (1 : 5000) (Sigma, USA). The immunogens were developed with ECL reagents A and B (TIANGEN, China,) according to the manufacturer's instructions.

### 2.5. Immunization and Challenge

The female BALB/c mice were randomly divided into five groups (26 mice in each group). The animals were subcutaneously injected with 100 *μ*g rTgTrxLp + rTgENO2 (G1), rTgTrxLp (G2), or rTgENO2 (G3) proteins emulsified in 100 *μ*L Freund's complete adjuvant (FCA), respectively. Two weeks following the primary immunization, mice in G1, G2, and G3 were inoculated with the same dose of each antigen plus incomplete Freund's adjuvant, respectively. The mice injected with equal adjuvant (G4) or PBS alone (G5) served as negative controls.

Fifteen mice in each group were subjected to acute infection by the intraperitoneal administration with 10^3^ *T. gondii *tachyzoites of GJS strain at 2 weeks after the third immunization. The challenged mice were monitored daily until total mortality. Six mice from each group were orally challenged with 10 cysts of* T. gondii* PRU strain at 2 weeks after the final immunization. Thirty days later, the animals were sacrificed to examine the number of brain cysts.

### 2.6. Collection of the Sera and Lymphocyte Samples

The blood samples of mice from all the groups were collected from the tail vein prior to each vaccination. The sera were harvested by centrifugation at 2000 ×g for 20 min and stored at −20°C until assayed for antibody titers and cytokines.

Two weeks after the final immunization, 3 mice per group were sacrificed to aseptically harvest the spleen. The spleens were pooled and filtered through a nylon membrane to obtain the splenocytes. Then, the cells were purified using erythrocyte lysis buffer (Solarbio, China) to remove the red blood cells. Subsequently, the harvested splenocytes were resuspended in DMEM medium supplemented with 10% fetal calf serum (FCS) and were used for the analysis of lymphocyte proliferation and the percentage of CD4^+^ and CD8^+^ T cells.

### 2.7. IgG ELISA

The specific humoral immune responses, anti-rTgTrxLp, rTgENO2, or the mixtures, were evaluated by ELISA using SBA Clonotyping System-HRP Kit (Southern Biotech Co., Ltd, Birmingham, USA), according to the manufacturer's instructions. The preparation of rTgTrxLp and rTgENO2 proteins was adjusted to 50 *μ*g/mL. The 96-well microtiter plates were coated with 100 *μ*L of each protein at 37°C for 2 h. 100 *μ*L of the prepared serum samples (1 : 10 dilution) from mice of each group was the added and incubated at RT for 1 h. The secondary antibody, goat anti-mouse HRP-IgG (Sigma), at 1 : 250 was incubated on the plate at RT for 1 h. After 3 rinses, the plates were visualized by incubating with substrate solution (pH 4.0) (1.05% citrate substrate buffer; 1.5% ABTS; 0.03% H_2_O_2_) for 15 min in dark, and then the reaction was stopped with 2 M H_2_SO_4_. The absorbance of each well was measured at 450 nm. All estimations were performed in triplicate.

### 2.8. Lymphocyte Proliferation Assays by MTS

The purified splenocytes per group were stimulated with the corresponding antigens (CAS) and concanavalin A (ConA, Sigma) after the density of cells was adjusted to 2 × 10^5^. The cells cocultured with medium alone served as the negative control. Four days later, the proliferation activity was measured by MTS method (Promega, USA). The stimulation index (SI) was calculated using the formula OD_490 CAS_/OD_490 M_ : OD_490 ConA_/OD_490 M_.

### 2.9. Flow Cytometry Analysis

The purified splenocytes resuspended in DMEM medium plus 10% FCS were incubated with fluorescently in-labeled anti-mouse IgG antibodies, including PE-CD3, APC-CD4, and FITC-CD8 (BioLegend, USA) for 30 min at 4°C. After PBS washes, the cells were fixed with FACS buffer (1% FCS plus 0.1% sodium azide in PBS) and 2% paraformaldehyde, under dim light. Data were collected and analyzed by System II software (Coulter).

### 2.10. Cytokine Assays

The collected sera from mice in each group were used to examine the levels of IL-2, IL-4, and IFN-*γ* in flat-bottom 96-well microtiter plates at 1 : 10 dilution. The detection was performed using commercial ELISA kits according to the manufacturer's instructions (BioLegend, USA). The data from three independent experiments were analyzed.

### 2.11. Statistical Analysis

One-way ANOVA was used for comparing the differences in antibody responses, percentages of CD4^+^ and CD8^+^ T cells, and the reduction in brain cysts. The difference of each variable in lymphoproliferation assays between the two groups was calculated by the *t*-test. The difference in survival time was calculated by the chi-square test. The figures were prepared by the GraphPad Prism statistical program, version 5.0 (San Diego, CA, USA). A value of *P* < 0.05 was considered significant.

## 3. Results

### 3.1. Identification of rTgTrxLp and rTgENO2 Proteins by SDS-PAGE and Western Blot

**Figure 1:**
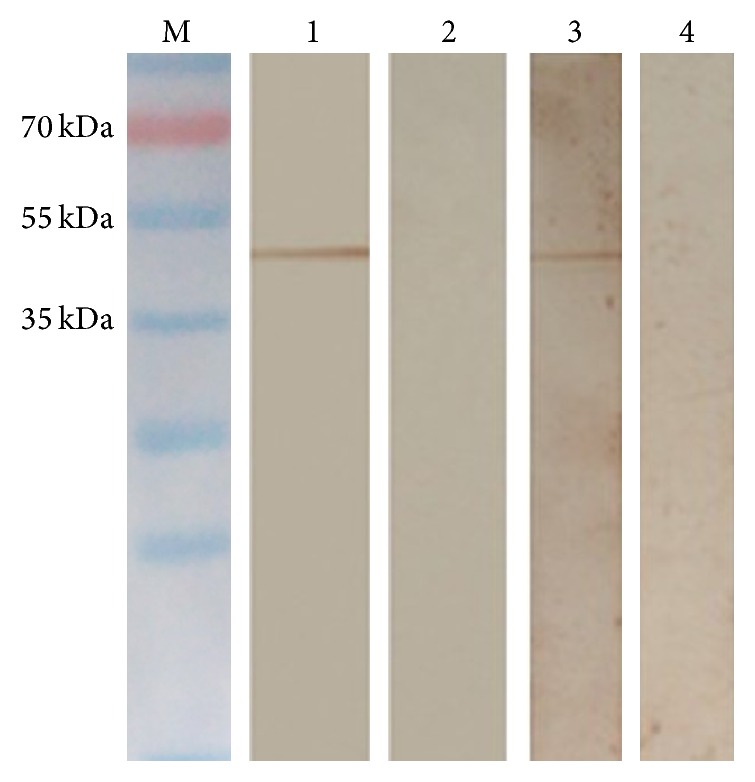
Identification of rTgTrxLp and rTgENO2 proteins with sera from pigs infected with* Toxoplasma gondii* GJS strain by Western blot. M: protein molecular weight; lanes 1 and 3: rTgTrxLp and rTgENO2 identified by sera from pigs infected with* T. gondii* GJS strain; lanes 2 and 4: rTgTrxLp and rTgENO2 reacted with* T. gondii* negative sera from pigs.

After respective transformation of pET-TrxLp and pET-ENO2 constructs into* E. coli* BL21 (DE3), the recombinant bacteria were lysed and proteins resolved using SDS-PAGE and stained with Coomassie Brilliant Blue. The rTgTrxLp and rTgENO2 proteins were identified as approximately 49 kDa, which coincided with the correspondingly theoretical molecular mass (Figure 1). The Western blot results showed that rTgTrxLp and rTgENO2 proteins could be identified by the sera from swine that was infected with* T. gondii*, as assessed by the positive band at nearly 49 kDa (Figure 1).

### 3.2. Humoral Immune Responses

**Figure 2:**
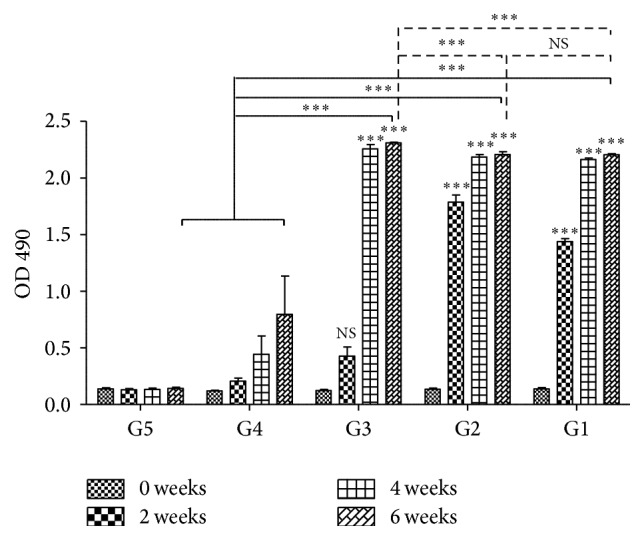
IgG antibodies induced by rTgTrxLp + rTgENO2 (G1), rTgTrxLp (G2), rTgENO2 (G3), adjuvant (G4), or PBS alone (G5) in the sera of mice at 0, 2, and 8 weeks. Each bar represents the mean OD (±SE, *n* = 3). ^*∗∗∗*^ *P* < 0.0001, NS: not significant compared to controls.

To analyze the specific humoral immune responses induced by various vaccines, the serum samples collected prior to each immunization were examined by ELISA (Figure 2). The results revealed that the IgG antibody elicited by each protein vaccine was continuously increased with successive immunization and reached a maximum level at 2 weeks after the final immunization [*F*(4,25) = 43.29, *P* < 0.0001] compared with that in the control groups. However, after the third immunization, the significantly highest IgG levels were detected in mice from G3 as compared to that in mice from G1 and G2 [*F*(2,15) = 14.87, *P* = 0.0003]. No statistically significant differences in levels of IgG antibodies in the sera from controls at 2 weeks after the last immunization were observed [*t*(10) = 1.937, *P* = 0.082].

### 3.3. Splenocyte Proliferation

**Figure 3:**
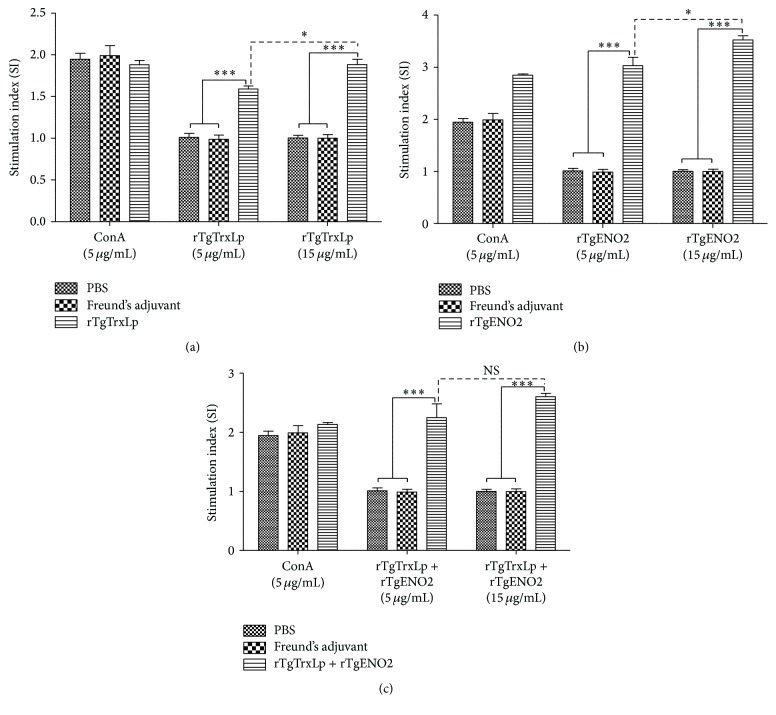
Splenocyte proliferation responses in immunized mice. ^*∗*^ *P* < 0.05, ^*∗∗∗*^ *P* < 0.0001, NS: not significantly represented splenocytes from mice immunized with rTgTrxLp (a), rTgENO2 (b), or rTgTrxLp + rTgENO2 (c) that was stimulated with 5 *µ*g/mL and 15 *µ*g/mL of each protein compared with those in mice from Freund's adjuvant and PBS. Each bar represents the mean stimulation index (± SE, *n* = 3).

The proliferation of splenocytes stimulated by antigens or ConA was examined using the MTS assay. The splenocytes from mice in G1, G2, and G3 were significantly proliferative after stimulation by the corresponding antigen proteins compared to that from mice in G4 and G5 (*P* < 0.0001, Figure 3). The levels of SI were significantly elevated with the increased concentration of rTgENO2 [*t*(4) = 2.782, *P* = 0.0497] and rTgTrxLp [*t*(4) = 4.056, *P* = 0.0154]. However, no significant difference was detected in the splenocytes in mice from G1 after coculturing with 15 *μ*g/mL and 5 *μ*g/mL rTgTrxLp + rTgENO2, respectively [*t*(4) = 1.490, *P* = 0.210].

### 3.4. Flow Cytometry Analysis

**Figure 4:**
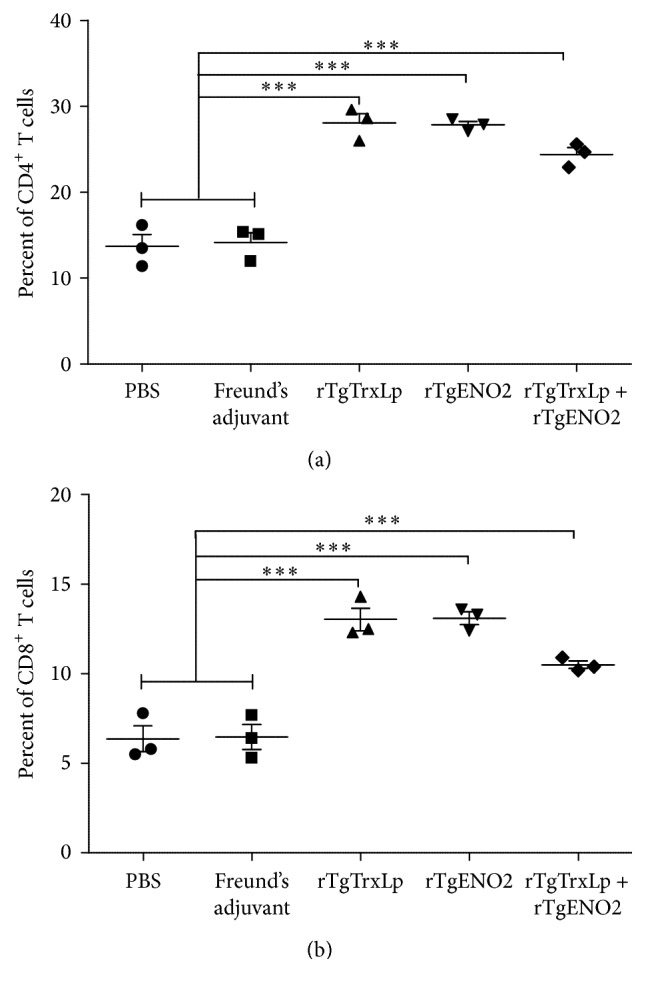
Percentages of CD4^+^ (a) and CD8^+^ (b) T cells in mice.   ^*∗∗∗*^ *P* < 0.001; NS: not significant compared to controls. Mice that received PBS and Freund's adjuvant were treated as controls in statistical analysis.

Percentages of CD4^+^ and CD8^+^ T cells in mice from each group were examined by flow cytometric analysis for the specific surface marker. The percentages of CD4^+^ T cells in total splenocytes in mice from G1 [24.4%  ±  1.37%, *F*(2,6) = 29.33, *P* = 0.0008], G2 [28.07%  ±  1.86%, *F*(2,6) = 46.90, *P* < 0.0002], or G3 [27.83%  ±  0.7%, *F*(2,6) = 59.04, *P* = 0.0001] were significantly higher than those in the controls, which varied from 13 to 14.17% (Figure 4(a)). Compared with the control groups, remarkably high levels of CD8^+^ T cells in mice from the G1 [*F*(2,6) = 15.95, *P* = 0.004], G2 [*F*(2,6) = 31.13, *P* = 0.0007], and G3 [*F*(2,6) = 39.46, *P* = 0.0004] groups were detected compared to that in the controls (Figure 4(b)).

### 3.5. Cytokine Assays

**Figure 5:**
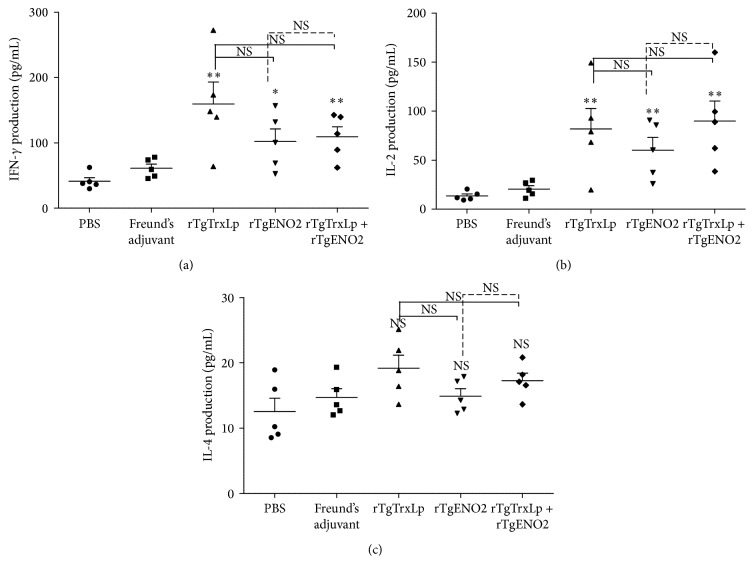
Cytokine production in sera of mice immunized with rTgTrxLp + rTgENO2, rTgTrxLp, and rTgENO2 compared to that in controls. (a) Levels of IFN-*γ*; (b) levels of IL-2; (c) levels of IL-4. ^*∗*^ *P* < 0.05 and ^*∗∗*^ *P* < 0.01; NS: not significant compared to controls. Mice that received PBS and Freund's adjuvant were treated as controls in statistical analysis. Each bar represents the mean OD (±SE, *n* = 5).

The cytokines in serum samples from each group were quantified by ELISA. As shown in Figure 5, levels of IFN-*γ* in mice that received rTgTrxLp [*F*(2,12) = 9.982, *P* = 0.0028], rTgENO2 [*F*(2,12) = 6.518, *P* = 0.0121], or rTgTrxLp + rTgENO2 [*F*(2,12) = 12.06, *P* = 0.0013] were significantly increased as compared to that in the controls. Also, the levels of IL-2 were significantly elevated in mice immunized with rTgTrxLp [*F*(2,12) = 9.394, *P* = 0.0035], rTgENO2 [*F*(2,12) = 10.51, *P* = 0.0023], or rTgTrxLp + rTgENO2 [*F*(2,12) = 12.24, *P* = 0.0013] as compared to that in the controls. However, any substantial differences in IL-4 were not seen among the groups (*P* = 0.07).

### 3.6. Protection against Acute and Chronic* T. gondii* Infection

**Figure 6:**
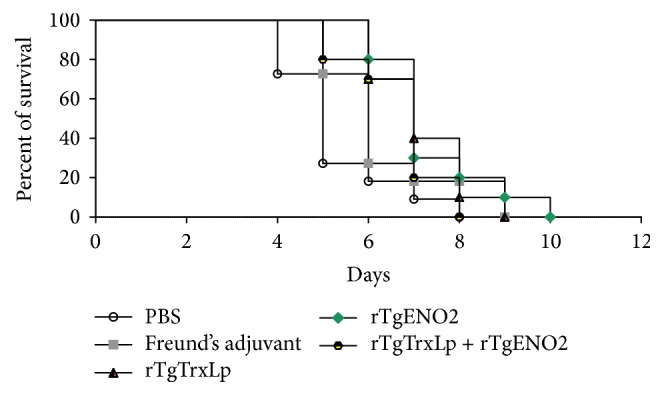
Survival rates of mice immunized with various protein vaccines followed by challenge. BALB/c mice were challenged with 1 × 10^3^ tachyzoites of* Toxoplasma gondii* GJS strain, 2 weeks after the final immunization.

As shown in Figure 6, the acute* T. gondii* infection in mice from G1 (8.38 ± 4.57 d), G2 (8 ± 2.77 d), and G3 (7.4 ± 1.81 d) survived significantly longer than the infected mice from G4 (6.78 ± 2.08 d) and G5 (6.38 ± 4.65 d) (*χ* ^2^ = 9.687, df = 4, *P* = 0.046).

**Figure 7:**
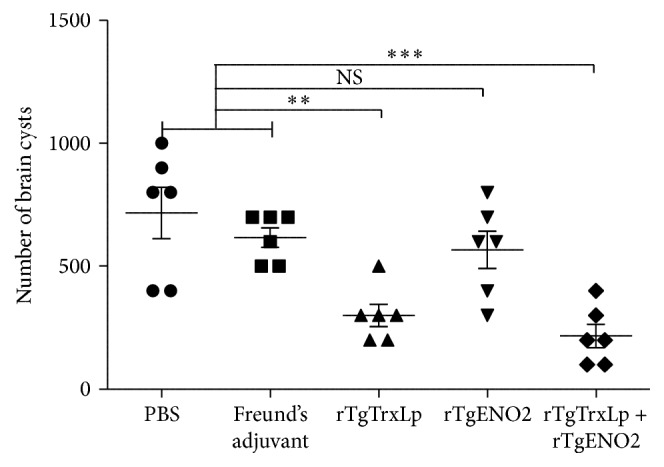
Number of tissue cysts per brain, after* Toxoplasma gondii* challenge, in mice from all groups. Mice immunized with rTgTrxLp + rTgENO2, rTgTrxLp, and rTgENO2 were challenged with 10 tissue cysts of* Toxoplasma gondii* PRU strain, 2 weeks after the final booster. ^*∗∗*^ *P* < 0.01 and ^*∗∗∗*^ *P* < 0.0001; NS: not significant compared to controls. Mice that received PBS and Freund's adjuvant were treated as controls in statistical analysis. Each bar represents the mean number (±SE, *n* = 6).

To evaluate whether rTgTrxLp, rTgENO2, and rTgTrxLp + rTgENO2 proteins could generate protective immunity against chronic* T. gondii* infection, mice from each group were challenged with 10 cysts of* T. gondii* PRU strain, following which the number of brain cysts was counted 30 days after challenged with infection. The number of brain cysts in mice vaccinated with rTgTrxLp (300 ± 109.54) [*F*(2,15) = 9.752, *P* = 0.0019] and rTgTrxLp + rTgENO2 (216.67 ± 116.90) [*F*(2,15) = 14.16, *P* = 0.0004] was significantly lower than that in the controls (Figure 7). However, the brain cysts in mice that received rTgENO2 (566.67 ± 109.54) were not significantly different from that in the controls [*F*(2,15) = 0.955, *P* = 0.407]. The brain cysts in mice from G1, G2, and G3 were reduced to 69.77%, 58.14%, and 20.93%, respectively, compared to that in the mice from G5.

## 4. Discussion

The immunogenicity of thioredoxin and enolase proteins from several protozoa and helminths has been well-evaluated, and both proteins were considered as potential vaccine candidates against those pathogens' infection [24, 25]. In the present study, mice immunized with rTgTrxLp and rTgENO2 proteins, either alone or in combination, induced strong humoral and cellular immune responses with significant longer survival time and lower brain cyst loadings after acute and chronic infection compared to that of the controls. This phenomenon indicated that the two antigens would be further used in the development of epitope peptide-based vaccines against* T. gondii* infection.

T cell-mediated immune response, especially the cytotoxic activity of CD8^+^ T cells, is critical for mediating resistance to* T. gondii* infection [26]. Herein, the T cell subclasses were stained by the surface markers, CD4 and CD8 molecules, and were analyzed by flow cytometry. The increased levels of CD4^+^ and CD8^+^ T cell in the mice from G1, G2, and G3 compared to that in controls would be essential for chronic toxoplasmosis.

During the acute stage of* T. gondii* infection, IFN-*γ*-mediated immune responses play a major role in resistance to the proliferation of tachyzoites [27–29]. After immunization with rTgTrxLp, rTgENO2, and rTgTrxLp + rTgENO2 proteins, the levels of IFN-*γ* were significantly higher than those in the controls, which would result in longer survival and lower numbers of brain cysts. IL-2 is another important cytokine that can stimulate CD8^+^ T cell proliferation after antigen presentation and also play the crucial role in the development of CD8^+^ T cells [30]. The levels of IL-2 in mice vaccinated with rTgTrxLp, rTgENO2, and rTgTrxLp + rTgENO2 proteins were significantly higher than that in the controls, which would also contribute to the protective efficacy against acute and chronic infection.* T. gondii* infection could induce Th1-dominant immune responses [31]. The increased level of IFN-*γ* and IL-2 in mice immunized with rTgTrxLp and rTgENO2 indicated that both the proteins could induce a Th1-biased immune response.

IL-4 exerts diverse functions in regulating the proliferation and differentiation of activated B cells [32] and is recognized as the Th2-type cytokine. Herein, the secretion of IL-4 in mice immunized with rTgTrxLp, rTgENO2, and rTgTrxLp + rTgENO2 proteins was not significantly higher than that in the controls.

IgGs were also considered critical in controlling the acute infection by* T. gondii* through opsonizing the parasite for phagocytosis and activating the classical complement pathway [33]. The immunization of mice with rTgTrxLp, rTgENO2, or rTgTrxLp + rTgENO2 could induce significantly high levels of IgG antibodies than that in the controls (*P* < 0.001), which would contribute to the strong protective efficacy against* T. gondii* infection. The results were in agreement with previous studies of vaccinating mice with plasmids coding ROM4, ROM5, [20] and CDPK3 [34], as well as the recombinant ROM1 [35], ROP18 [21], and ROP38 [36–38].

In conclusion, the present study demonstrated that both the rTgTrxLp and rTgENO2 proteins can generate humoral and cellular immune responses in a mouse model and can significantly prolong the survival time and reduce brain cyst number. Mice immunized with the combination of the two proteins showed the longest survival time (8.38 ± 4.57 d) and the lowest brain cyst number (69.77%) when comparing controls and mice immunized with a single protein. These results indicated that rTgTrxLp and rTgENO2 proteins could be used as potential candidates in the development of multicomponent vaccines against toxoplasmosis.

## References

[1] Dubey J. P. (2010). *Toxoplasmosis of Animals and Humans*.

[2] Robert-Gangneux F., Dardé M.-L. (2012). Epidemiology of and diagnostic strategies for toxoplasmosis. *Clinical Microbiology Reviews*.

[3] Elmore S. A., Jones J. L., Conrad P. A., Patton S., Lindsay D. S., Dubey J. P. (2010). *Toxoplasma gondii*: epidemiology, feline clinical aspects, and prevention. *Trends in Parasitology*.

[4] Dubey J. P., Lago E. G., Gennari S. M., Su C., Jones J. L. (2012). Toxoplasmosis in humans and animals in Brazil: high prevalence, high burden of disease, and epidemiology. *Parasitology*.

[5] Zhou P., Chen Z. G., Li H.-L. (2011). *Toxoplasma gondii* infection in humans in China. *Parasites & Vectors*.

[6] Dubey J. P. (2009). Toxoplasmosis in pigs-the last 20 years. *Veterinary Parasitology*.

[7] Dubey J. P., Hill D. E., Jones J. L. (2005). Prevalence of viable *Toxoplasma gondii* in beef, chicken, and pork from retail meat stores in the United States: risk assessment to consumers. *Journal of Parasitology*.

[8] Innes E. A., Bartley P. M., Maley S. W., Wright S. E., Buxton D. (2007). Comparative host-parasite relationships in ovine toxoplasmosis and bovine neosporosis and strategies for vaccination. *Vaccine*.

[9] Fajardo H. V., D'Ávila S., Bastos R. R. (2013). Seroprevalence and risk factors of toxoplasmosis in cattle from extensive and semi-intensive rearing systems at Zona da Mata, Minas Gerais state, Southern Brazil. *Parasites and Vectors*.

[10] Verin R., Mugnaini L., Nardoni S. (2013). Serologic, molecular, and pathologic survey of *Toxoplasma gondii* infection in free-ranging red foxes (*Vulpes vulpes*) in central Italy. *Journal of Wildlife Diseases*.

[11] Feo S., Arcuri D., Piddini E., Passantino R., Giallongo A. (2000). ENO1 gene product binds to the c-myc promoter and acts as a transcriptional repressor: relationship with Myc promoter-binding protein 1 (MBP-1). *FEBS Letters*.

[12] Subramanian A., Miller D. M. (2000). Structural analysis of *α*-enolase: mapping the functional domains involved in down-regulation of the c-*myc* protooncogene. *The Journal of Biological Chemistry*.

[13] Mouveaux T., Oria G., Werkmeister E. (2014). Nuclear glycolytic enzyme enolase of *Toxoplasma gondii* functions as a transcriptional regulator. *PLoS ONE*.

[14] Kanzok S. M., Fechner A., Bauer H. (2001). Substitution of the thioredoxin system for glutathione reductase in *Drosophila melanogaster*. *Science*.

[15] Ahn J. H., Choi J. H., Song J. M. (2011). Increase in Trx2/Prx3 redox system immunoreactivity in the spinal cord and hippocampus of aged dogs. *Experimental Gerontology*.

[16] Drechsel D. A., Patel M. (2010). Respiration-dependent H_2_O_2_ removal in brain mitochondria via the thioredoxin/peroxiredoxin system. *The Journal of Biological Chemistry*.

[17] Liu J., Wetzel L., Zhang Y. (2013). Novel thioredoxin-like proteins are components of a protein complex coating the cortical microtubules of *Toxoplasma gondii*. *Eukaryotic Cell*.

[18] Decoster A., Darcy F., Capron A. (1988). Recognition of *Toxoplasma gondii* excreted and secreted antigens by human sera from acquired and congenital toxoplasmosis: identification of markers of acute and chronic infection. *Clinical and Experimental Immunology*.

[19] Prigione I., Facchetti P., Lecordier L. (2000). T cell clones raised from chronically infected healthy humans by stimulation with *Toxoplasma gondii* excretory-secretory of the clones and implications for vaccine development. *The Journal of Immunology*.

[20] Zhang N.-Z., Xu Y., Wang M. (2015). Protective efficacy of two novel DNA vaccines expressing *Toxoplasma gondii* rhomboid 4 and rhomboid 5 proteins against acute and chronic toxoplasmosis in mice. *Expert Review of Vaccines*.

[21] Qu D., Han J., Du A. (2013). Enhancement of protective immune response to recombinant *Toxoplasma gondii* ROP18 antigen by ginsenoside Re. *Experimental Parasitology*.

[22] Qu D., Han J., Du A. (2013). Evaluation of protective effect of multiantigenic DNA vaccine encoding MIC3 and ROP18 antigen segments of *Toxoplasma gondii* in mice. *Parasitology Research*.

[23] Wang Y., Wang G., Zhang D., Yin H., Wang M. (2013). Identification of novel B cell epitopes within *Toxoplasma gondii* GRA1. *Experimental Parasitology*.

[24] Anugraha G., Madhumathi J., Prince P. R. (2015). Chimeric epitope vaccine from multistage antigens for lymphatic filariasis. *Scandinavian Journal of Immunology*.

[25] Gupta R., Kumar V., Kushawaha P. K. (2014). Characterization of glycolytic enzymes—rAldolase and rEnolase of *Leishmania donovani*, identified as Th1 stimulatory proteins, for their immunogenicity and immunoprophylactic efficacies against experimental visceral leishmaniasis. *PLoS ONE*.

[26] Parker S. J., Roberts C. W., Alexander J. (1991). CD8+ T cells are the major lymphocyte subpopulation involved in the protective immune response to *Toxoplasma gondii* in mice. *Clinical and Experimental Immunology*.

[27] Suzuki Y., Orellana M. A., Schreiber R. D., Remington J. S. (1988). Interferon-*γ*: the major mediator of resistance against *Toxoplasma gondii*. *Science*.

[28] Sturge C. R., Benson A., Raetz M. (2013). TLR-independent neutrophil-derived IFN-*γ* is important for host resistance to intracellular pathogens. *Proceedings of the National Academy of Sciences of the United States of America*.

[29] Ely K. H., Kasper L. H., Khan I. A. (1999). Augmentation of the CD8+ T cell response by IFN-gamma in IL-12-deficient mice during *Toxoplasma gondii* infection. *The Journal of Immunology*.

[30] Zhang N., Bevan M. J. (2011). CD8^+^ T cells: foot soldiers of the immune system. *Immunity*.

[31] Suzuki Y., Sa Q., Gehman M., Ochiai E. (2011). Interferon-gamma- and perforin-mediated immune responses for resistance against *Toxoplasma gondii* in the brain. *Expert Reviews in Molecular Medicine*.

[32] Bessieres M.-H., Swierczynski B., Cassaing S. (1997). Role of IFN-*γ*, TNF-*α*, IL4 and IL 10 in the regulation of experimental *Toxoplasma gondii* infection. *Journal of Eukaryotic Microbiology*.

[33] Sayles P. C., Gibson G. W., Johnson L. L. (2000). B cells are essential for vaccination-induced resistance to virulent *Toxoplasma gondii*. *Infection and Immunity*.

[34] Zhang N.-Z., Huang S.-Y., Zhou D.-H. (2013). Protective immunity against *Toxoplasma gondii* induced by DNA immunization with the gene encoding a novel vaccine candidate: calcium-dependent protein kinase 3. *BMC Infectious Diseases*.

[35] Li J., Han Q., Gong P. (2012). *Toxoplasma gondii* rhomboid protein 1 (TgROM1) is a potential vaccine candidate against toxoplasmosis. *Veterinary Parasitology*.

[36] Xu Y., Zhang N.-Z., Wang M. (2015). A long-lasting protective immunity against chronic toxoplasmosis in mice induced by recombinant rhoptry proteins encapsulated in poly (lactide-co-glycolide) microparticles. *Parasitology Research*.

[37] Zhang N.-Z., Chen J., Wang M., Petersen E., Zhu X.-Q. (2013). Vaccines against *Toxoplasma gondii*: new developments and perspectives. *Expert Review of Vaccines*.

[38] Zhang N.-Z., Wang M., Xu Y., Petersen E., Zhu X.-Q. (2015). Recent advances in developing vaccines against *Toxoplasma gondii*: an update. *Expert Review of Vaccines*.

